# A Prospective Cohort Longitudinal Study of Human Acute Babesiosis: Quality of Life and Severity of Symptoms Through 1-Year Follow-up

**DOI:** 10.1093/ofid/ofaf668

**Published:** 2025-12-05

**Authors:** Rudline Zamor, Xiaoyue Zhang, Pooja Lamba, Brigitte Maczaj, Sarath G Nath, Aikaterini Papamanoli, Bennadette Maramara, Michael Lum, Atul Pradhan, Christine Li, Victoria Bateman, Jie Yang, Charles K Vorkas, Eric D Spitzer, Dana G Mordue, Luis A Marcos

**Affiliations:** Division of Infectious Diseases, Department of Medicine, Stony Brook University, Stony Brook, New York, USA; Biostatistical Consulting Core, School of Medicine, Stony Brook University (State University of New York), Stony Brook, New York, USA; Division of Infectious Diseases, Department of Medicine, Stony Brook University, Stony Brook, New York, USA; Division of Infectious Diseases, Department of Medicine, Stony Brook University, Stony Brook, New York, USA; Division of Infectious Diseases, Department of Medicine, Stony Brook University, Stony Brook, New York, USA; Division of Infectious Diseases, Department of Medicine, Stony Brook University, Stony Brook, New York, USA; Division of Infectious Diseases, Department of Medicine, Stony Brook University, Stony Brook, New York, USA; Division of Infectious Diseases, Department of Medicine, Stony Brook University, Stony Brook, New York, USA; Division of Infectious Diseases, Department of Medicine, Stony Brook University, Stony Brook, New York, USA; Division of Infectious Diseases, Department of Medicine, Stony Brook University, Stony Brook, New York, USA; Division of Infectious Diseases, Department of Medicine, Stony Brook University, Stony Brook, New York, USA; Biostatistical Consulting Core, School of Medicine, Stony Brook University (State University of New York), Stony Brook, New York, USA; Department of Family, Population and Preventive Medicine, Stony Brook University, Stony Brook, New York, USA; Division of Infectious Diseases, Department of Medicine, Stony Brook University, Stony Brook, New York, USA; Department of Microbiology and Immunology, Stony Brook University, Stony Brook, New York, USA; Department of Pathology, Stony Brook University, Stony Brook, New York, USA; Department of Pathology, Microbiology, and Immunology, New York Medical College, Valhalla, New York, USA; Division of Infectious Diseases, Department of Medicine, Stony Brook University, Stony Brook, New York, USA; Department of Microbiology and Immunology, Stony Brook University, Stony Brook, New York, USA

**Keywords:** *Babesia*, immunocompromised, quality of life, severity of symptoms

## Abstract

**Background:**

Babesiosis, caused by the parasitic blood-borne piroplasm *Babesia microti*, is emerging in the Northern hemisphere. We aimed to study long-term symptoms of patients with *B microti* infection in New York.

**Methods:**

A prospective longitudinal cohort study of human babesiosis was conducted at Stony Brook University Hospital. Inclusion criteria were age ≥18 years with positive blood smear for *Babesia* spp. Symptoms were assessed in patients at presentation and at 1, 6, and 12 months by 3 validated surveys: a visual analog scale, a quality of life (QOL) questionnaire, and the 36-Item Short Form Survey (SF-36).

**Results:**

In total, 38 patients with acute *B microti* infection (26% female; age range, 54–73 years) were enrolled from 2020 to 2022. Compared with baseline, the visual analog scale total symptom scores (with high scores representing worse status) significantly decreased at 6-month follow-up for the immunocompetent (n = 9; *P* < .001) and immunocompromised groups (n = 6; *P* < .001). Scores remained significantly higher in the immunocompromised group (ratio, 2.6; *P* = .045). At 1-year follow-up, the scores in the 2 groups tended to be similar (ratio, 0.9; *P* = .82). Within QOL concept scores (with low scores representing worse status), physical functioning significantly increased after 6 months of follow-up in both cohorts (immunocompetent, n = 10 [*P* = .004]; immunocompromised, n = 5 [*P* = .008]) but was still significantly lower in the immunocompromised group at that time (ratio, 0.7; *P* < .001). By the 12-month follow-up, physical functioning scores in the 2 groups appeared to converge, though the difference remained borderline significant (ratio, 0.9; *P* = .06).

**Conclusions:**

The time to convalescence was similar among patients with babesiosis, though immunocompromised patients tended to have more prolonged symptoms and worsened QOL after babesiosis at 1-year follow-up, compared with immunocompetent patients.

Tick-borne diseases are a growing public health concern in the United States, particularly in the Northeast [1–7]. *Babesia microti*, a parasitic blood-borne piroplasm, causes the disease babesiosis in humans [8, 9]. Patients typically acquire this infection after a bite from *Ixodes scapularis* ticks, though transmission can occur through blood transfusions in rare cases [10, 11]. Individuals who contract this disease may experience clinical manifestations ranging from asymptomatic or mild symptoms [12] to severe or fulminant courses for those of older age and depressed immune status [13, 14]. Individuals identified as immunocompromised are more at risk of having long-term symptoms from babesiosis due to persistence or relapsing infection or due to conditions similar to malaria that may cause chronic symptoms, such as fever, fatigue, and chill caused by ongoing hemolysis, but which usually resolve after appropriate treatment [7, 15]. Patient quality-of-life (QOL) surveys are commonly used to assess patient symptoms during disease states [16]. However, the impact of babesiosis infection on long-term QOL has not been systematically evaluated in previous studies [1–5].

Prolonged symptoms following an infection are not uncommon. For example, after the acute phase of infection in Lyme disease (LD), caused by spirochetes in the *Borrelia burgdorferi* sensu lato complex, treatment trials in patients with erythema migrans have estimated that approximately 10%–20% experience persistent symptoms, such as fatigue, generalized musculoskeletal pain, or cognitive difficulties lasting >6 months. This condition is referred to as posttreatment LD syndrome [17, 18]. This syndrome has been found to be more prevalent for disseminated or late-stage LD cases (20.9% [95% confidence interval (CI), 6.8%–64.4%]) than for cases limited to erythema migrans without evidence of disseminated infection (5.9% [2.7%–12.9%), suggesting that disease severity may be associated with long-term symptoms [17].

Similarly, persistent symptoms have been observed after severe acute respiratory syndrome coronavirus 2 (SARS-CoV-2) infection, a condition referred to as postacute sequalae of coronavirus disease 2019 (COVID-19) [19]. This syndrome is estimated to affect approximately 18.9% of individuals infected with COVID-19 [20], and while reduced circulating serotonin levels have been implicated, much of the underlying pathogenesis remains unknown [21]. In areas endemic for tick-borne diseases, clinicians frequently encounter patients with a history of LD or babesiosis who report persistent symptoms [18]. In the case of babesiosis, some of these prolonged symptoms may be due to relapsing infection, particularly in immunocompromised individuals [15]. However, there is a limited number of prospective longitudinal cohort studies investigating whether this intraerythrocytic infection can persist in patients with ongoing symptoms or how commonly such prolonged symptoms occur in this population.

To our knowledge, the current study is the first prospective longitudinal cohort study that aims to examine the frequency and severity of symptoms and the QOL after treatment in patients with microscopy-proved acute human babesiosis stratified by immunocompromised status before infection and up to 1 year of follow-up in an endemic area of Suffolk County, Long Island, New York. Our hypothesis is that immunocompromised patients are at higher risk of prolonged symptoms after treatment.

## METHODS

### Study Design

A longitudinal prospective cohort study was conducted in Long Island, New York, to enroll individuals with acute *Babesia* infections from 2020 to 2022. Participants were scheduled for follow-up assessments at 1, 6, and 12 months after enrollment. Follow-up appointments were scheduled at the time of enrollment by the research coordinator, who contacted patients up to 3 times by phone, in addition to 1 email reminder, to encourage attendance at study visits. Stony Brook University Hospital (SBUH) is a 600-bed tertiary medical center located in Suffolk County, New York, the county with the highest number of reported *Babesia* cases in New York State (135 of 589 [23%] in 2022) [22].

### Inclusion Criteria

Adult individuals (≥18 years of age) who visited the emergency department (ED) at SBUH or SBUH clinics and presented with symptoms of babesiosis were tested with a blood smear and a polymerase chain reaction (PCR) test. Those who had a positive blood smear for intraerythrocytic parasites consistent with *Babesia* spp were enrolled into the cohort study; subsequently, and per state policy, these patients all had a confirmed PCR *B microti* result by the New York State Department of Health. Immunocompromised patients were defined as individuals with asplenia, active cancer, a history of transplantation, treatment with biologic agents (eg, anti-CD20), or chronic corticosteroid use. Informed consent was obtained from all study participants (Stony Brook University Institutional Review Board approval no. 1210472).

### Data Collection

This study used primarily longitudinal scoring data, with demographic and clinical information collected at enrollment (baseline). Participants’ symptoms and health status were evaluated using visual analog scale (VAS) and the 36-Item Short Form Survey (SF-36) (RAND), with scores collected at baseline and at 1, 6, and 12 months after enrollment at the Clinical Research Center of SBUH.

### VAS Assessment

Twelve symptoms were assessed using the VAS: loss of appetite, joint pain, cough, dizziness, fatigue, fever/chills, headache, muscle pains, nausea/vomiting, tingling/abnormal sensation, stiff neck, and difficulty concentrating/memory problems. This 8-cm VAS was completed at the time of each visit based on current symptoms ≥48 hours in duration. For any symptoms marked, a licensed healthcare provider would determine their potential cause. For each symptom, the score of each symptom ranged from 0 to 8, with higher scores indicating worse health status [23]. In statistical analysis, the number of symptoms and the total summed score were examined.

### QOL Assessment

Participants’ QOL was assessed through the SF-36, which addresses the following 8 health concepts: physical functioning, role limitation due to physical health, role limitation due to emotional problems, energy/fatigue, emotional well-being, social functioning, pain, and general health. Each concept was scored on a 0–100 scale, with higher scores representing better status. All patients completed on their own the questionnaire on QOL symptoms. In the subsequent analysis, concept scores were analyzed separately.

### *Babesia* PCR on Follow-up Visits

DNA extraction with *B microti* was performed as described elsewhere [24]. DNA was extracted from 200 µL of frozen blood stored at −80°C, using a GeneJET whole-blood genomic DNA purification kit (K0781; Thermo Scientific), per the manufacturer’s protocol. The 18S ribosomal RNA gene sequence of *B microti* was used to detect the presence of pathogen DNA in the blood. A primer/probe combination was used to detect the target gene using Bm18Sf-AACAGGCATTCGCCTTGAAT, Bm18Sr-CCAACTGCTCCTATTAACCATTACTCT, and 6FAM-CTACAGCATGGAATAATGA-MGBQ (probe), respectively [24]. The 20-µL PCR master mix consisted of 2X Taqman Universal PCR Master Mix (Applied Biosystems), 0.9 µmol/L Bm18Sf, 0.9 µmol/L Bm18Sr, and 0.2 µmol/L probe; 100 ng of total DNA per sample was used in individual PCR reactions carried out on a Roche LightCycler 480 instrument II. Cycling conditions were as follows: 50°C for 2 minutes, 95°C for 10 minutes, and 40 cycles of denaturation at 95°C for 15 seconds and annealing at 59°C for 60 seconds (with signal acquisition at the end of each cycle). Quantitative genomic DNA from *B microti* (PRA-398DQ; American Type Culture Collection) was used as a positive control. The detection limit was 0.5 pg or 1.4 copies of *B microti* DNA.

### Statistical Analysis

In general results (Supplementary Tables 1 and 2), bivariate analysis was applied to categorical variables using χ^2^ or Fisher exact tests and to continuous variables using Student *t* tests. In the descriptive analysis, patients were stratified into 2 groups based on baseline immune status prior to *B microti* infection—immunocompromised or immunocompetent—and demographic and scoring variables were compared between the 2 groups. Categorical data were summarized as counts and proportions, while continuous data were presented as medians with interquartile ranges (IQRs; 25th to 75th percentiles). To compare categorical variables between groups, χ^2^ tests with exact *P* values based on Monte-Carlo simulations were used; for continuous variables, Wilcoxon rank sum tests were performed. To analyze the longitudinal trajectory of symptom scores and health quality scores, linear mixed-effects models were used to examine the scores across 4 time points (at baseline and 1, 6, and 12 months). Log transformation on outcome was applied to meet the normality assumption. Fixed effects in the models included immune group and time point. To account for the relationship between age and symptoms, a categorical variable for age (<65 vs ≥65 years) was a covariate in all models. An interaction term between immune status and time was added to model the potential differences in score changes between immunocompromised and immunocompetent patients.

Based on the Akaike information criteria, the covariance structure to model correlation among longitudinal measurement from the same patient was selected from the following: compound symmetry, unstructured, autoregressive with order 1, Toeplitz, heterogeneous compound symmetry, heterogeneous autoregressive with order 1, and heterogeneous Toeplitz. All model assumptions have been checked through residual diagnosis, such as relevant Q-Q plots for normality assumption. Subgroup analysis was performed by excluding patients who were coinfected to assess the robustness of the findings within a more homogeneous population. To address potential bias due to missing data, a sensitivity analysis was conducted in which all patients lost to follow-up were conservatively assumed to be completely cured, with a symptom score of 0. To accommodate the log transformation in the linear mixed-effects model, a small constant (0.001) was added to these 0 values. Differences were considered statistically significant at *P* < .05, and analyses were performed using SAS 9.4 software (SAS Institute).

## RESULTS

### Patients

A total of 38 adult patients with acute babesiosis (26% female; age range, 54–73 years; 42% aged ≥65 years) with peripheral blood smear positive for *Babesia* were enrolled between 2020 to 2022. All samples were subsequently confirmed by PCR to be *B microti*. Two cases were diagnosed in the outpatient clinics, the rest in the ED. All 36 patients who presented to the ED were hospitalized. Seven patients (18.4%) were identified as immunocompromised due to asplenia (n = 2), active cancer (n = 2), or transplantation, use of biologics (eg, anti-CD20), or chronic steroid use (n = 3), and the remaining 31 (81.6%) were immunocompetent. Five of the immunocompromised patients were prescribed prolonged treatment (≥6 weeks) with atovaquone and azithromycin, compared with to 7–10 days of the same antimicrobials for immunocompetent patients (Supplementary Table 1). Most patients were white (n = 23 [60.5%]); 3 (7.9%) were African American, 11 (28.9%) were Hispanics, and 1 (2.6%) was Asian. Clinical manifestations, treatment regimen and laboratory results are presented in Supplementary Tables 1 and 2. Some patients were diagnosed with tick-borne coinfections with *B microti*; 11 were diagnosed with a LD coinfection. Three patients had a follow-up test with results that remained positive at the 1-month mark, 12 had a negative/nonreactive result, and 23 were not tested at this point (Supplementary Table 2). Patients were not tested for LD during the 6- or 12-month follow-up.

### Longitudinal Symptoms

VAS results were examined among patients with an initial assessment completed at enrollment. Of the 38 enrolled patients, 33 completed their baseline VAS assessment; 7 (21.2%) were identified as immunocompromised, and 26 (78.8%) as immunocompetent (Table 1). Five patients were excluded. Four did not have any VAS value, and 1 did not have a baseline value but had values at other time points; However, that patient only reported 2 symptoms, and the total symptom score was 0. Immunocompromised patients tended to be older than immunocompetent ones (median age, 74 vs 59.5 years, respectively; *P* = .18). Four immunocompromised (57.1%) and 10 immunocompetent (38.5%) patients were aged ≥65 years, but the difference was not statistically significant (*P* = .42).

**Table 1.** Patient Characteristics by Immune Status for Visual Analog Scale Analysis

Characteristic	Total (N = 33)	Patients, No. (%)^a^		*P* Value^b^
		Immunocompromised (n = 7 [21.2%])	Immunocompetent (n = 26 [78.8%])	
Age, median (IQR), y	60 (54–73)	74 (54–78)	59.50 (54–73)	.18
Age group, y				
<65	19 (57.58)	3 (42.86)	16 (61.54)	.42
≥65	14 (42.42)	4 (57.14)	10 (38.46)	
Sex				
Female	10 (30.30)	3 (42.86)	7 (26.92)	.65
Male	23 (69.70)	4 (57.14)	19 (73.08)	
Race/ethnicity				
African American	3 (9.09)	0 (0.00)	3 (11.54)	.64
Asian	1 (3.03)	0 (0.00)	1 (3.85)	
Hispanic	8 (24.24)	1 (14.29)	7 (26.92)	
White	21 (63.64)	6 (85.71)	15 (57.69)	

Abbreviation: IQR, interquartile range.

^a^Data represent no. (column %) unless otherwise specified.

^b^ *P* values based on χ^2^ test with Monte-Carlo simulation for categorical and Wilcoxon rank sum test for continuous variables.

Twenty-five patients completed the 1-month, 15 the 6-month, and 12 the 12-month visit. Follow-up compliance 71.4% for the immunocompromised and 76.9% for the immunocompetent group at 1 month, 85.7% and 34.6%, respectively, at 6 months, and 57.1% and 30.8% at 12 months.

At enrollment, all patients reported ≥2 types of symptoms on the VAS (Supplementary Table 3). After 1 month of treatment, the proportion dropped to 60% (3 of 5) in the immunocompromised and 50% (10 of 20) in the immunocompetent group (*P* = .69). By 6 months, 66.7% of the immunocompromised patients (4 of 6) reported ≥2 of the 12 symptoms, compared with 22.2% of the immunocompetent ones (2 of 9) (*P* = .09). At 12 months, both groups had 25% of patients still reporting ≥2 symptoms (1 of 4 in the immunocompromised vs 2 of 8 in the immunocompetent group).

Immunocompromised patients reported a median 5 of symptoms (IQR, 3–7) at the time of initial diagnosis, while immunocompetent patients reported a median of 7 (6–9) at baseline (Supplementary Table 4). The total symptom score was higher for immunocompetent than for immunocompromised patients (median, 35.5 vs 22; *P* = .05). Among immunocompetent patients, the number of symptoms dropped to a median (IQR) of 1.5 (0.5–4.5) at 1 month and to 0 (0–1) at 6 months. In contrast, immunocompromised patients reported a median (IQR) of 4 symptoms (1–4) at 1 month, and 2.5 (1–4) at 6 months. The total severity score was higher in the immunocompromised than in the immunocompetent group at 1 month (median, 7 vs 4.74, respectively; *P* = .58) and at 6 months (5.75 vs 0; *P* = .11), corresponding with the greater number of symptoms in the immunocompromised group, though this difference did not reach statistical significance. Among all immunocompromised patients, symptoms at 6 months were similar in all groups (cancer, biologics, and asplenia; *P* = .8) (Supplementary Table 5).

Each symptom was reported at least once at baseline in both groups. The 3 most reported symptoms for the immunocompromised and immunocompetent groups were fatigue (in 100% vs 96.2%, respectively), decreased appetite (85.7% vs 84.6%), and fever (71.4% vs 92.31%). The symptoms with the lowest frequency for immunocompetent patients were tingling, stiff neck, and nausea/vomiting (at 26.9%, 30.8%, and 34.6% respectively); for the immunocompromised group the low-frequency symptoms were joint pain, tingling, and stiff neck at 14.3% each and nausea/vomiting and muscle pains at 28.6% each (Table 2).

**Table 2.** Number of Symptomatic Patients by Time Point for Specific Symptoms

Symptom	Time Point	Patients, No. Symptomatic/Total No. (%)		*P* Value^a^
		Immunocompromised	Immunocompetent	
Fatigue	Baseline	7/7 (100)	25/26 (96.2)	>.99
	mo 1	3/5 (60)	14/20 (70)	>.99
	mo 6	4/6 (66.7)	1/9 (11.1)	.09
	mo 12	1/4 (25)	4/8 (50)	.58
Appetite	Baseline	6/7 (85.7)	22/26 (84.6)	>.99
	mo 1	3/5 (60)	5/20 (25)	.29
	mo 6	2/6 (33.3)	0/9 (0)	.14
	mo 12	0/4	1/8 (12.5)	>.99
Fever	Baseline	5/7 (71.4)	24/26 (92.3)	.19
	mo 1	1/5 (20)	1/20 (5)	.38
	mo 6	0/6	0/9	…
	mo 12	0/4	2/8 (25)	.52
Cough	Baseline	4/7 (57.1)	13/26 (50)	>.99
	mo 1	3/5 (60)	3/20 (15)	.0705
	mo 6	0/6	1/9 (11.1)	>.99
	mo 12	0/4	1/8 (12.50)	>.99
Dizzy	Baseline	4/7 (57.1)	16/26 (61.5)	>.99
	mo 1	2/5 (40)	7/20 (35)	>.99
	mo 6	2/6 (33.3)	1/9 (11.1)	.53
	mo 12	0/4	1/8 (12.5)	>.99
Difficulty concentrating	Baseline	4/7 (57.1)	15/26 (57.7)	>.99
	mo 1	3/5 (60)	6/20 (30)	.31
	mo 6	2/6 (33.3)	2/9 (22.2)	>.99
	mo 12	1/4 (25)	2/8 (25)	>.99
Headache	Baseline	3/7 (42.9)	19/26 (73.1)	.18
	mo 1	0/5	2/20 (10)	>.99
	mo 6	0/6	0/9	…
	mo 12	0/4	1/8 (12.50)	>.99
Muscle pain	Baseline	2/7 (28.6)	14/26 (53.8)	.40
	mo 1	0/5	5/20 (25)	.55
	mo 6	1/6 (16.7)	1/9 (11.1)	>.99
	mo 12	0/4	1/8 (12.5)	>.99
Nausea/vomiting	Baseline	2/7 (28.6)	9/26 (34.6)	>.99
	mo 1	0/5	1/20 (5)	>.99
	mo 6	0/6	0/9	…
	mo 12	0/4	1/8 (12.5)	>.99
Joint pain	Baseline	1/7 (14.3)	12/26 (46.1)	.20
	mo 1	0/5	1/20 (5)	>.99
	mo 6	1/6 (16.7)	1/9 (11.1)	>.99
	mo 12	1/4 (25)	2/8 (25)	>.99
Tingling	Baseline	1/7 (14.3)	7/26 (26.9)	.66
	mo 1	0/5	4/20 (20)	.55
	mo 6	2/6 (33.3)	1/9 (11.1)	.53
	mo 12	1/4 (25)	1/8 (12.5)	>.99
Stiff neck	Baseline	1/7 (14.3)	8/26 (30.8)	.64
	mo 1	0/5	6/20 (30)	.28
	mo 6	0/6	0/9	…
	mo 12	0/4	2/8 (25)	.52

^a^ *P* values based on χ^2^ test with Monte-Carlo simulation.

From adjusted linear mixed-effects model results, both immunocompromised and immunocompetent patients had symptom scores significantly drop after 1 month of treatment as expected, with progressive decrease in symptom scoring at 6 and 12 months. At 1 month, the total severity score in the immunocompromised group was 0.28 times lower than that at baseline (95% CI, .08–.93; *P* = .04). The score dropped to 0.21 times lower at 6 months (95% CI, .09–.47; *P* < .01) and 0.09 times lower at 12 months (.03–.27; *P* < .01). In the immunocompetent group, the total severity score dropped to 0.13 (95% CI, .07–.25), 0.06 (.03–.11), and 0.07 (.03–.16) times lower (each *P* < .01) at 1, 6, and 12 months, respectively (Supplementary Table 6). There was no significant difference in the total severity score at baseline between the 2 immune groups. However, at 6 months, the score was significantly higher—2.64 times (95% CI, 1.02–6.81; *P* = .045)—among immunocompromised than among immunocompetent patients (Supplementary Table 7). Further analysis on longitudinal symptoms, excluding those identified with coinfection, is provided in Supplementary Table 9.

### QOL Findings

**Figure 1.**
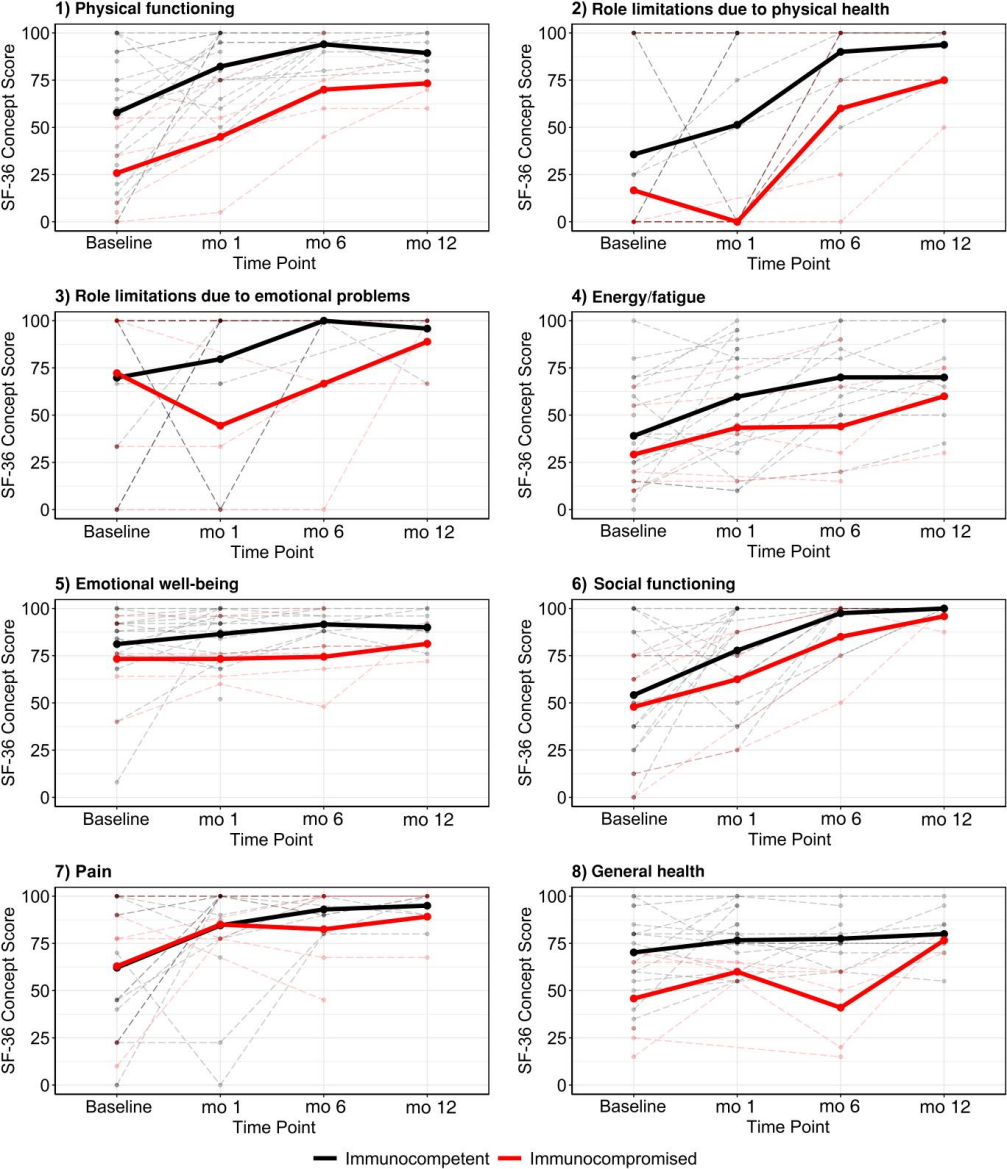
Concept scores for the 36-Item Short Form Health Survey (SF-36) at 4 time points stratified by immune group. The thin lines were collected scores from individual patients, and the thick lines were group averages.

**Figure 2.**
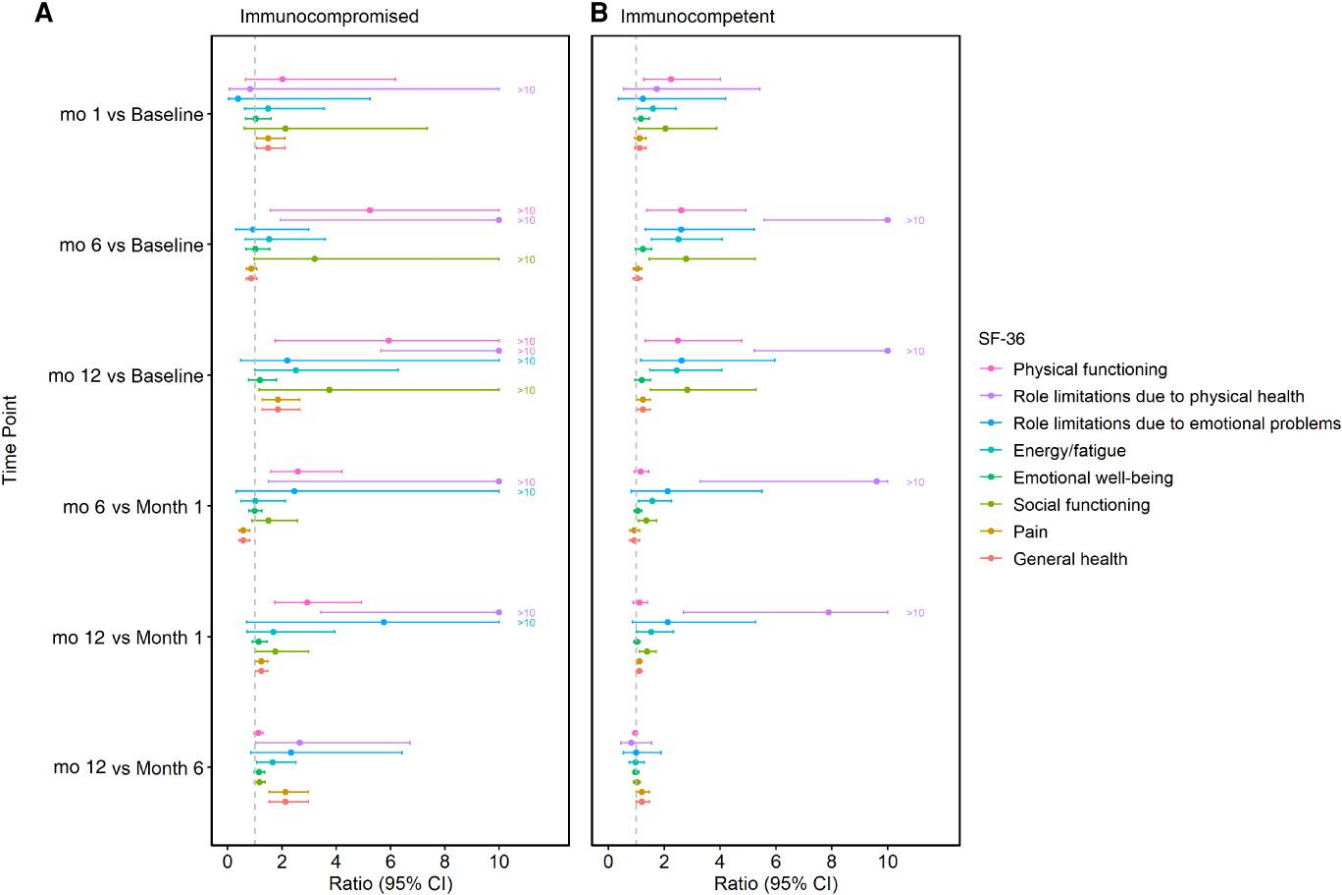
Estimated ratios of 36-Item Short Form Health Survey (SF-36) concept scores across time points within each immune group, based on linear mixed-effects models. For better visualization, point estimates and upper confidence interval (CI) bounds exceeding 10 were truncated at 10.

For SF-36 concepts, 29 of the 38 enrolled patients completed the initial survey (immunocompromised, n = 6 [20.7%]) (Supplementary Table 8). Nine patients were excluded, as they did not have available SF-36 values at the analysis time point. Note that, for the 29 patients who remained in the final analysis, 2 did not have baseline SF-36 values; their first survey was submitted at visit 2 (1-month follow-up). The group averages for SF-36 concept scores are shown in Figure 1. At baseline, the physical functioning score did not differ significantly between immunocompromised and immunocompetent groups (ratio, 0.36 [95% CI, .09–1.45]; *P* = .15). However, both groups experienced a significant increase in physical functioning scores at month 6. The immunocompromised group showed a 5.24-fold increase (95% CI, 1.58–17.40; *P* < .01), while the immunocompetent group had a 2.61-fold increase (1.38–4.93; *P* < .01). Nonetheless, physical functioning scores were still lower in the immunocompromised group at month 6 (ratio, 0.73 [95% CI, .62–.86]; *P* < .01). Results for other 7 types of concepts are listed in Figure 2 and Supplementary Table 9.

### *Babesia* PCR

Of the 38 patients enrolled, 27 completed ≥1 follow-up visit for blood samples at month 1, 6, or 12 after initial enrollment. Of these 27 patients, 7 were immunocompromised. PCR for *B microti* was performed on these follow-up convalescent samples. Of 7 immunocompromised patients, 6 had follow-up blood samples. One of 6 had a persistently positive PCR result for *B microti* at 1-month follow-up. Of the 19 immunocompetent patients, 1 was also found to have a positive PCR result at 1-month follow-up (Supplementary Table 1). All PCR results were negative at 6-month and 12-month follow-up for both immune groups (Supplementary Table 1 and Table 3).

**Table 3.** *Babesia* Test Results in 7 Immunocompromised Patients

Patient No. and Immunocompromised Status	Length of Admission, d	Parasitemia (%)		PCR *Babesia* PCR Results (Follow-up)	Treatment	Symptoms at 6 mo
		Initial	Final			
1. Chronic leukemia	8	2.5	<0.1	Negative at 2 and 6 mo	Atovaquone (8 d), azithromycin (8 d), and doxycycline (5 d)	Yes
2. Asplenia	13	6.3	No parasite seen	Positive at 1 mo; negative at 6 and 12 mo	Atovaquone (6 wk), azithromycin (6 wk), and doxycycline (10 d)	Yes
3. B-cell lymphoma	14	2.4	No parasite seen	Negative at 1, 6, and 12 mo	Atovaquone (>6 mo) and azithromycin (>6 mo)	Yes
4. LGL leukemia	4	0.9	No parasite seen	Negative at 1 and 6 mo	Atovaquone (14 d), azithromycin (14 d), and doxycycline (21 d)	Yes
5. Lymphoma	5	1.3	0.1	Negative at 1, 6, and 12 mo	Atovaquone (6 wk), azithromycin (6 wk), and doxycycline (14 d)	No
6. Asplenia	9	12.5	No parasite seen	Positive at 1 mo; negative at 6 and 12 mo	Atovaquone (6 wk), azithromycin (6 wk), doxycycline (7 d), clindamycin (9 d), and quinine (9 d)	Yes
7. Anti-CD10 therapy	7	1.5	0.4	NA	Atovaquone (6 wk), azithromycin (6 wk), and doxycycline (3 wk)	NA

Abbreviations: LGL, large granular lymphocytic; NA, not available; PCR, polymerase chain reaction.

## DISCUSSION

This 1-year longitudinal cohort study of human babesiosis is among the most comprehensive clinical examinations of long-term symptoms in this emerging tick-borne disease. Historically, long-term symptoms caused by tick-borne diseases are often reported for LD, and patient QOL is not often subjected to further analysis [17]. In the current study, we enrolled 36 patient with moderate-severe babesiosis who presented to the ED and were admitted to general medicine floors and 2 patients who were diagnosed in outpatient clinics (Supplementary Figure 1 and Supplementary Table 1). To our knowledge, this is largest study to date that assesses long-term postbabesiosis symptoms for up to 1 year.

Although symptoms improved for all patients during follow-up, more rapid convalescence was observed in the immunocompetent group. This suggests that those with immunocompromised status may have had more severe initial disease, predisposing them to prolonged convalescence. The immunocompromised group had a trend for higher APACHE-II scores (Supplementary Table 2) and had significantly higher parasitemia (3.9% vs 2.1%; *P* = .02). Our study excluded the possibility that prolonged symptoms were related to persistent parasitemia. Two of 6 immunocompromised patient had a positive PCR result at their 1-month follow-up. Our results indicate that immunocompromised individuals were more likely to report persistent symptoms after infection despite prolonged courses of antibiotics and prompt clearance of parasitemia. Whether the persistence of symptoms may be related to anemia recovery, cytokine dysregulation, autoantibodies, persistent endothelial injury, or another proinflammatory pathway remains an open question. Nonetheless, current evidence suggests that persistent parasitemia is unlikely to be the primary cause of prolonged symptoms. Further research is needed to elucidate the underlying mechanisms.

Among all symptoms, fatigue was identified as the most common symptom in both groups and with up to 1-year follow-up, in line with previous reports of fatigue being a lingering symptom in babesiosis [8]. Fatigue after infections is commonly reported (with LD, Epstein-Barr virus, and COVID-19, among others) [25, 26]. However, the exact mechanism of this phenomenon remains unknown and is hypothesized to be related to chronic immune activation [27]. Postinfectious sequelae have recently emerged as a significant complication of SARS-CoV-2 infection, commonly referred to as *long COVID*. Longitudinal cohort studies have demonstrated that long COVID can present with fatigue, musculoskeletal pain, and neurological symptoms that may persist for more than a year [28]. Proposed underlying mechanisms include the production of autoantibodies, such as those targeting mitochondria and neurons, which have been associated with decreased functional status and more severe respiratory symptoms [29]. Similarly, in babesiosis, prolonged and persistent symptoms tend to occur in patients with more severe disease, particularly those with high parasitemia or immunocompromised status. A comparable pattern is observed in LD, where individuals with more severe manifestations, such as neurological involvement, are more likely to experience persistent symptoms.

Regarding QOL, immunocompromised reported worse QOL symptoms during 1-year follow-up, compared with immunocompetent individuals. QOL impairment has previously been used as an outcome measure following LD [17], but it has not been reported in postbabesiosis cases. The SF-36 assessment tool is a promising instrument to assess patient QOL [16–18]. Our study showed similar improvement in QOL between groups, though immunocompromised patients tended to have lower QOL scores.

These findings may have important implications for clinical practice. When monitoring patients for posttreatment symptoms of babesiosis, healthcare providers may be inclined to attribute persistent fatigue to ongoing parasitemia. However, our study demonstrated that follow-up PCR testing of blood samples remained negative for up to 1 year, without evidence of relapsing disease. In immunocompromised patients, clinicians should remain cautious, as symptoms may persist longer after antibiotic treatment in these patients than in immunocompetent individuals. In addition, it is an open question whether comorbid conditions, prolonged anemia, inflammatory pathways, or other mechanisms are associated with persistent symptoms in these cases. Notably, parasitemia does not appear to be present in patients with prolonged symptoms. Therefore, clinicians should recognize that additional antibiotic therapy is not indicated.

We acknowledge the limitations of our study. The analysis did not include a control group without babesiosis to assess background symptom prevalence. Fatigue and musculoskeletal pain are common in the general population; therefore, without appropriate controls, attributing their persistence specifically to babesiosis should be approached with caution. Although objective clinical comorbid conditions were assessed, our results rely on subjective reporting that may introduce confounding variables. While evidence of coinfection was demonstrated in some patients with babesiosis and LD, the sample size was not sufficient to perform further analysis for coinfection. Furthermore, there were no significant differences in baseline characteristics between patients with or without coinfection.

To test the robustness of our findings, we excluded the 11 coinfected patients from the analysis. Results from the linear mixed-effects model evaluating the log-transformed VAS total symptom scores remained consistent with those obtained from the full cohort of 33 participants (Supplementary Table 10). Because LD is known to cause long-term symptoms, some coinfected patients may have posttreatment LD syndrome rather than symptoms attributable solely to babesiosis. This potential confounding bias highlights the need for further studies to differentiate the contributions of each infection. In endemic areas, however, a positive serological result for LD is not uncommon and may not necessarily indicate an acute coinfection with *Babesia*. Regardless, our findings (excluding coinfected patients) remain consistent, indicating that some symptoms, such as fatigue, can persist in patients whose babesiosis parasitemia has cleared.

Certain symptoms noted at 6- or 12-month follow-up may result from distinct causes not captured by our dataset. Loss to follow-up may also have affected results during follow-up. It may be inferred that the higher attrition rate for immunocompetent group is due to patients no longer having symptoms, potentially creating an attrition bias for the VAS and QOL analysis. To address this limitation, we first compared baseline characteristics between patients who were lost to follow-up and those who remained in the study. These comparisons were conducted based on 2 definitions of study retention: (1) patients who completed the full 12-month follow-up and (2) patients who remained in the study only from baseline to month 1 (considered as those who experienced rapid symptom resolution). Baseline VAS symptom scores did not differ significantly between these groups. Race was the only characteristic showing a difference, which we attribute to the small number of African American and Asian patients (n = 4), all of whom were immunocompetent.

In addition, we performed a sensitivity analysis dealing with the missing values and assuming that patients lost to follow-up were cured. The results remained consistent with our main findings: VAS total symptom scores were significantly decreased at 6-month follow-up in both the immunocompetent (*P* < .001) and immunocompromised (*P* = .003) groups, and scores remained significantly higher in the immunocompromised group at 6 months (*P* < .001) (Supplementary Tables 11–13).

In conclusion, we provide the first longitudinal analysis of patient symptoms following acute babesiosis stratified by baseline immunocompromised state, for up to 1 year after acute diagnosis. Our results show persistent symptoms in some patients. It remains unknown whether this could be related to the acute immune response after primary infection or postinfectious inflammatory sequelae. Future studies that include longitudinal analysis of immune parameters—such as monocyte, natural killer, T-cell, and B-cell frequency and activation and effector functions—will enable testing of the hypothesis that dysregulated immune responses are correlated with symptom persistence following microbiologic clearance of primary *B microti* infection. Our ongoing work seeks to test plausible mechanistic pathways, including the role of innate and innatelike lymphocytes, *Babesia* antigen–specific T cells, prolonged proinflammatory cytokine secretion, and autoantibody responses during acute infection and recovery.

## Supplementary Material

ofaf668_Supplementary_Data

## Supplementary Data

Supplementary materials are available at *Open Forum Infectious Diseases* online. Consisting of data provided by the authors to benefit the reader, the posted materials are not copyedited and are the sole responsibility of the authors, so questions or comments should be addressed to the corresponding author.
