# Supplementary material for: A Prospective Cohort Longitudinal Study of Human Acute Babesiosis: Quality of Life and Severity of Symptoms Through 1-Year Follow-up
Source: Open Forum Infect Dis. 2025 Dec 5;12(12):ofaf668. doi: 10.1093/ofid/ofaf668 (PMC12679593; doi:10.1093/ofid/ofaf668)
Supplement: ofaf668_Supplementary_Data [file ofaf668_supplementary_data.docx]

**Supplementary Materials**

This section presents supplemental tables regarding the research study: A Prospective Cohort Longitudinal Study of Human Acute Babesiosis: Quality of Life and Severity of Symptoms up to 1-year Follow Up.

**Contents**:

Supplementary Figure 1: STROBE Flow Diagram of Participants

Supplementary Table 1: Demographics, clinical characteristics, and treatment of patients with *B. microti* infection

Supplementary Table 2: Descriptive table of patients’ Clinical Values

## Supplementary Table 3: Number of symptomatic patients at each time point

## Supplementary Table 4: Number of symptom(s) and severity score of symptomatic subjects at each time point

Supplementary Table 5. Persistent symptoms at 6 months in Immunocompromised patients (n=7).

## Supplementary Table 6: Estimated ratio of VAS total severity score across time points within each immune group based on linear mixed effect model

## Supplementary Table 7: Estimated ratio of VAS total severity score between immunocompromise group and immunocompetent group at each time point based on linear mixed effect model

## Supplementary Table 8: Descriptive table of patients’ characteristics by immune status for SF-36 analysis

## Supplementary Table 9: Estimated ratio of SF-36 concept scores between immune groups at each time point based on linear mixed effect models

## Supplementary Table 10: Estimated mean values of VAS total score at each time point stratified by immune status based on linear mixed-effects model after excluding patients who were co-infected at baseline

Supplementary Table 11: Type 3 p-values of fixed effects in a linear mixed-effect models fitting log-transformed VAS total score assuming patients lost to follow-up were cured (VAS=0)

Supplementary Table 12: Estimated mean values of VAS total score at each time point stratified by immune status based on linear mixed-effects model

Supplementary Table 13: Estimated ratio of VAS total score across time points within each immune group based on linear mixed-effects model

Supplementary Figure 1: STROBE Flow Diagram of Participants


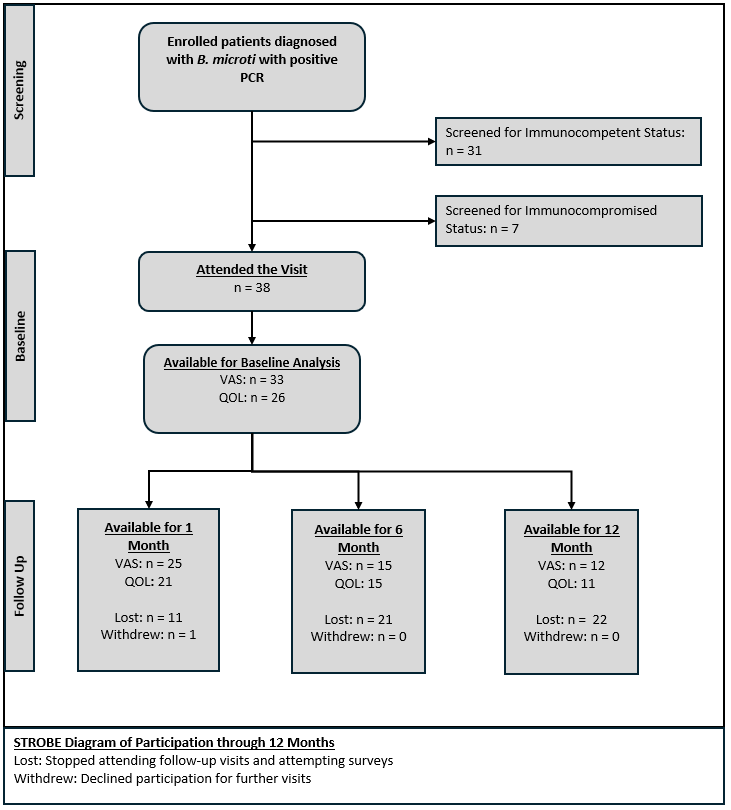


Supplementary Table 1: Demographics, clinical characteristics, and treatment of patients with *B. microti* infection

| **Characteristics** | **Total (N= 38)** | **Immunocompetent  (N = 31)** | **Immunocompromised  (N= 7)** | **P-value** |
| --- | --- | --- | --- | --- |
| Admission | | | | |
| General Medicine (GM) | 36 (94.74%) | 29 (96.77%) | 7 (100%) | 0.6344 |
| Length of GM Stay | 5.14 days | 4.62 days | 8.57 days | - |
| ICU Admission | 5 (13.16%) | 4 (12.90%) | 1 (14.29%) | 0.9228 |
| Outpatient Clinics | 2 (5.26%) | 2 (6.45%) | 0 (0%) | 0.4957 |
| Treatment | | | | |
| Atovaquone | 38 (100%) | 31 (100%) | 7 (100%) | - |
| Azithromycin | 38 (100%) | 31 (100%) | 7 (100%) | - |
| Doxycycline | 35 (92.11%) | 29 (93.55%) | 6 (85.71%) | 0.4930 |
| Quinine | 1 (2.63%) | 0 (0%) | 1 (14.29%) | 0.0353 |
| Clindamycin | 2 (5.26%) | 1 (3.23%) | 1 (14.29%) | 0.2430 |
| Comorbidities | | | | |
| No PMHx | 9 (23.68%) | 9 (29.03%) | 0 (0%) | 0.1074 |
| Diabetes Mellitus | 6 (15.79%) | 5 (16.13%) | 1 (14.29%) | 0.9053 |
| Hypertension | 14 (36.84%) | 12 (38.71%) | 2 (28.57%) | 0.6201 |
| Leukemia/Lymphoma | 4 (10.53 %) | 0 (0%) | 4 (57.14%) | <0.0001 |
| Asplenia | 2 (5.26%) | 0 (0%) | 2 (28.57%) | 0.0026 |
| Immunosuppression Medication | 1 (2.63%) | 0 (0%) | 1 (14.29%) | 0.0353 |
| Note: For categorical variables, the column percentages were reported. | | | | |

Supplementary Table 2: Descriptive table of patients’ Clinical Values

| **A. Clinical Values of Babesiosis Patients** | | | | |
| --- | --- | --- | --- | --- |
| **Clinical Values** | **Total (N=38)** | **Immunocompetent (N=31)** | **Immunocompromised (N=7)** | **P-value** |
| Initial Hemoglobin | 11.58 ±1.79 | 11.81 ± 1.76 | 10.54 ± 1.56 | 0.0876 |
| Initial White Blood Cells | 5.96 ± 2.50 | 5.91 ± 2.00 | 6.18 ± 4.29 | 0.8002 |
| Initial Platelet Count | 93.18 ± 51.25 | 93.55 ± 54.72 | 91.57 ± 34.90 | 0.9279 |
| Parasitemia (1st recorded) | 2.41 ± 2.46 | 2.07 ± 1.82 | 3.91 ± 1.58 | 0.0185 |
| Parasitemia (Max) | 2.63 ± 2.86 | 2.29 ± 2.38 | 4.13 ± 4.34 | 0.1255 |
| Temp (Max C) | 37.92 ± 0.99 | 37.95 ± 0.93 | 37.79 ± 1.30 | 0.6939 |
| Charlson Comorbidities Index | 76.58 ± 27.26 | 83.10 ± 18.79 | 47.71 ± 37.28 | 0.0007 |
| APACHE II Scores | 7.5 ± 4.64 | 6.39 ± 3.74 | 12.43 ± 5.29 | 0.001 |
| Note: Data reported as mean ± standard deviation. | | | | |
| **B. Screening of Babesia *microti* (qPCR)** | | | | |
| **Follow Up** | **Total (N=38)** | **Immunocompetent (N=31)** | **Immunocompromised (N=7)** |  |
| Babesia PCR Month 1 | 24 Tested: 3 Positive 21 Negative | 19 Tested:  1 positive 18 Negative | 5 Tested: 2 positive 3 Negative* |  |
| Babesia PCR Month 6 | 15 Tested: 15 Negative | 9 Tested: 9 Negative | 6 Tested:  6 Negative |  |
| Babesia PCR Month 12 | 15 Tested: 15 Negative | 11 Tested: 11 Negative | 4 Tested: 4 Negative |  |
| Note: *One patient in the immunocompromised group had a test done at 2 months as indicated in Table 3. | | | | |
| **C. Screening of Lyme Test (2-tier testing method, CDC criteria)** | | | | |
|  | **Total (N=38)** | **Immunocompetent (N=31)** | **Immunocompromised (N=7)** |  |
| Lyme positive at initial visit | 11 (28.9) | 8 (29.03) | 3 (42.86) |  |
| Lyme positive at Month 1 | 3 (7.89) | 2 (6.45) | 1 (14.29) |  |
| Note: For categorical variables, the column percentages were reported. | | | | |

## Supplementary Table 3: Number of symptomatic patients at each time point

| **Time point** | **Immunocompromised** | **Immunocompetent** | **P-value^1^** |
| --- | --- | --- | --- |
| **For those with symptom(s)** | | |  |
| Baseline | 7/7 (100%) | 26/26 (100%) | . |
| Month 1 | 4/5 (80%) | 15/20 (75%) | 0.8149 |
| Month 6 | 5/6 (83.3%) | 4/9 (44.4%) | 0.1320 |
| Month 12 | 3/4 (75%) | 4/8 (50%) | 0.4076 |
| **For those with 2 or more symptoms** | | |  |
| Baseline | 7/7 (100%) | 26/26 (100%) | . |
| Month 1 | 3/5 (60%) | 10/20 (50%) | 0.6889 |
| Month 6 | 4/6 (66.7%) | 2/9 (22.2%) | 0.0852 |
| Month 12 | 1/4 (25%) | 2/8 (25%) | 1.0000 |
| 1: p-values were based on Chi-squared test with Monte-Carlo simulation.  Note: data were presented as no./No. (%). | | | |

## Supplementary Table 4: Number of symptom(s) and severity score of symptomatic subjects at each time point

| **Variable** | **Time point** | **Immunocompromised** | **Immunocompetent** | **P-value^1^** |
| --- | --- | --- | --- | --- |
| No. of Symptom(s) | Baseline | 5 (3, 7) | 7 (6, 9) | 0.1691 |
|  | Month 1 | 4 (1, 4) | 1.5 (0.5, 4.5) | 0.7295 |
|  | Month 6 | 2.5 (1, 4) | 0 (0, 1) | 0.0974 |
|  | Month 12 | 1 (0.5, 1.5) | 0.5 (0, 3) | 0.7877 |
| Total Severity Score | Baseline | 22 (20, 34) | 35.5 (29.25, 49) | 0.0450 |
|  | Month 1 | 7 (5, 11) | 4.75 (0.5, 12.25) | 0.5840 |
|  | Month 6 | 5.75 (2, 8) | 0 (0, 2.5) | 0.1132 |
|  | Month 12 | 2 (1, 3) | 0.5 (0, 7) | 0.7913 |
| 1: p-values were based on Wilcoxon rank-sum tests.  Note: data were from patients with symptom at each time point, and were represented as median (Q1, Q3). | | | | |
|  | | | | |

Supplementary Table 5. Persistent symptoms at 6 months in Immunocompromised patients (n=7).

| **SUBTYPE** | **N = 7** | **Symptoms at 6 months** | **P Value** |
| --- | --- | --- | --- |
| Malignancy | 2 | 2 (100.00) | 0.8187 |
| Biologics | 3 | 1 (33.33) |  |
| Asplenia | 2 | 2 (100.00) |  |

## Supplementary Table 6: Estimated ratio of VAS total severity score across time points within each immune group based on linear mixed effect model

|  | **Immunocompromised** | | **Immunocompetent** | |
| --- | --- | --- | --- | --- |
| **Time point** | **Estimated ratio**  **(95% CI)** | **P-value^1^** | **Estimated ratio**  **(95% CI)** | **P-value^1^** |
| Month 1 vs Baseline | 0.28 (0.08, 0.93) | 0.0375 | 0.13 (0.07, 0.25) | <.0001 |
| Month 6 vs Baseline | 0.21 (0.09, 0.47) | 0.0004 | 0.06 (0.03, 0.11) | <.0001 |
| Month 12 vs Baseline | 0.09 (0.03, 0.27) | <.0001 | 0.07 (0.03, 0.16) | <.0001 |
| Month 6 vs Month 1 | 0.75 (0.19, 2.86) | 0.6627 | 0.43 (0.19, 0.98) | 0.0459 |
| Month 12 vs Month 1 | 0.31 (0.07, 1.47) | 0.1371 | 0.55 (0.21, 1.43) | 0.2138 |
| Month 12 vs Month 6 | 0.42 (0.11, 1.52) | 0.1795 | 1.28 (0.49, 3.35) | 0.6109 |
| *: P-values were from Type III analysis based on a linear mixed-effect model with further adjustment of age group. | | | | |

## Supplementary Table 7: Estimated ratio of VAS total severity score between immunocompromise group and immunocompetent group at each time point based on linear mixed effect model

| **Immune Status** | **Time point** | **Estimated ratio**  **(95% CI)** | **P-value*** |
| --- | --- | --- | --- |
| Immunocompromised vs Immunocompetent | Baseline | 0.75 (0.46, 1.21) | 0.2261 |
|  | Month 1 | 1.53 (0.42, 5.52) | 0.5106 |
|  | Month 6 | 2.64 (1.02, 6.81) | 0.0453 |
|  | Month 12 | 0.86 (0.23, 3.24) | 0.8201 |
| *: P-values were from Type III analysis based on a linear mixed-effect model with further adjustment of age group. | | | |

## Supplementary Table 8: Descriptive table of patients’ characteristics by immune status for SF-36 analysis

| **Characteristics** | **Level** | **Total (N=29)** | **Immunocompromised (N=6, 20.7%)** | **Immunocompetent (N=23, 79.3%)** | **P-value** |
| --- | --- | --- | --- | --- | --- |
| Age (year) | Unit = 1 | 62 (54, 74) | 75.50 (62, 78) | 59 (52, 73) | 0.0707 |
| Age | <65 | 17 (58.62%) | 2 (33.33%) | 15 (65.22%) | 0.1940 |
|  | >=65 | 12 (41.38%) | 4 (66.67%) | 8 (34.78%) |  |
| Gender | Female | 8 (27.59%) | 3 (50.00%) | 5 (21.74%) | 0.3140 |
|  | Male | 21 (72.41%) | 3 (50.00%) | 18 (78.26%) |  |
| Race\ethnicity | African American | 2 (6.90%) | 0 | 2 (8.70%) | 0.3969 |
|  | Asian | 1 (3.45%) | 0 | 1 (4.35%) |  |
|  | Hispanic | 6 (20.69%) | 0 | 6 (26.09%) |  |
|  | White | 20 (68.97%) | 6 | 14 (60.87%) |  |
| Note: For categorical variables, the column percentages were reported; for continuous variables median (Q1, Q3) were reported. | | | | | |

## Supplementary Table 9: Estimated ratio of SF-36 concepts scores between immune groups at each time point based on linear mixed effect models

|  | **Immunocompromised vs Immunocompetent** | |
| --- | --- | --- |
| **Time point** | **Estimated ratio (95% CI)** | **P-value** |
| **Physical functioning** | | |
| Baseline | 0.36 (0.09, 1.45) | 0.1465 |
| Month 1 | 0.33 (0.18, 0.58) | 0.0004 |
| Month 6 | 0.73 (0.62, 0.86) | 0.0003 |
| Month 12 | 0.86 (0.74, 1.01) | 0.0582 |
| **Role limitations due to physical health** | | |
| Baseline | 0.31 (0.04, 2.41) | 0.2556 |
| Month 1 | 0.15 (0.01, 2.30) | 0.1676 |
| Month 6 | 0.24 (0.07, 0.79) | 0.0205 |
| Month 12 | 0.78 (0.61, 1.00) | 0.0501 |
| **Role limitations due to emotional problems** | | |
| Baseline | 1.08 (0.19, 6.05) | 0.9255 |
| Month 1 | 0.34 (0.04, 3.11) | 0.3232 |
| Month 6 | 0.39 (0.12, 1.22) | 0.1027 |
| Month 12 | 0.91 (0.72, 1.15) | 0.4058 |
| **Energy/fatigue** | | |
| Baseline | 0.73 (0.26, 2.07) | 0.5461 |
| Month 1 | 0.68 (0.26, 1.82) | 0.4337 |
| Month 6 | 0.45 (0.25, 0.79) | 0.0070 |
| Month 12 | 0.75 (0.48, 1.18) | 0.2081 |
| **Emotional well-being** | | |
| Baseline | 0.94 (0.58, 1.52) | 0.7942 |
| Month 1 | 0.83 (0.66, 1.04) | 0.1084 |
| Month 6 | 0.78 (0.66, 0.93) | 0.0063 |
| Month 12 | 0.93 (0.80, 1.08) | 0.3415 |
| **Social functioning** | | |
| Baseline | 0.72 (0.19, 2.73) | 0.6182 |
| Month 1 | 0.75 (0.42, 1.32) | 0.3041 |
| Month 6 | 0.83 (0.68, 1.02) | 0.0758 |
| Month 12 | 0.96 (0.91, 1.00) | 0.0603 |
| **Pain** | | |
| Baseline | 0.71 (0.18, 2.84) | 0.6235 |
| Month 1 | 0.72 (0.42, 1.24) | 0.2272 |
| Month 6 | 0.81 (0.65, 1.00) | 0.0486 |
| Month 12 | 0.96 (0.91, 1.00) | 0.0645 |
| **General health** | | |
| Baseline | 0.58 (0.40, 0.85) | 0.0069 |
| Month 1 | 0.78 (0.60, 0.99) | 0.0452 |
| Month 6 | 0.49 (0.34, 0.71) | 0.0004 |
| Month 12 | 0.87 (0.67, 1.14) | 0.3032 |
| *: P-values were from Type III analysis based on a linear mixed-effect model with further adjustment of age group | | |

## Supplementary Table 10: Estimated mean values of VAS total score at each time point stratified by immune status based on linear mixed-effects model after excluding patients who were co-infected at baseline

|  | **Estimated mean of VAS total score** | |
| --- | --- | --- |
| **Time point** | **Immunocompetent** | **Immunocompromised** |
| Baseline | 29.25 (21.43, 39.93) | 22.07 (11.39, 42.75) |
| Month 1 | 2.72 (1.29, 5.71) | 7.22 (1.64, 31.82) |
| Month 6 | 1.50 (0.77, 2.90) | 5.54 (2.29, 13.37) |
| Month 12 | 2.39 (0.89, 6.43) | 2.21 (0.82, 5.98) |

Supplementary Table 11: Type 3 p-values of fixed effects in a linear mixed-effect models fitting log-transformed VAS total score assuming patients lost to follow-up were cured (VAS=0)

| **Variable** | **Num DF** | **Den DF** | **F Value** | **P-value*** |
| --- | --- | --- | --- | --- |
| Immune group | 1 | 30 | 0.75 | 0.3929 |
| Time point | 3 | 46 | 38.93 | <.0001 |
| Immune group * Time point | 3 | 46 | 2.13 | 0.1091 |
| Age group | 1 | 30 | 3.26 | 0.0812 |
| *: P-values were from Type III analysis based on a linear mixed-effect model using Heterogeneous Compound Symmetry covariance structure. A small count (0.001) was added to zero values to avoid issue in log-transformation of VAS score. | | | | |

Supplementary Table 12: Estimated mean values of VAS total score at each time point stratified by immune status based on linear mixed-effects model

|  | **Estimated mean of VAS total score** | |
| --- | --- | --- |
| **Time point** | **Immunocompetent** | **Immunocompromised** |
| Baseline | 31.56 (25.26, 39.44) | 23.49 (15.41, 35.79) |
| Month 1 | 0.17 (0.03, 0.99) | 0.20 (0.01, 6.22) |
| Month 6 | 0.00 (0.00, 0.01) | 0.52 (0.04, 6.40) |
| Month 12 | 0.00 (0.00, 0.01) | 0.03 (0.00, 0.38) |

Supplementary Table 13: Estimated ratio of VAS total score across time points within each immune group based on linear mixed-effects model

|  | **Immunocompetent** | | **Immunocompromised** | |
| --- | --- | --- | --- | --- |
| **Time point** | **Estimated ratio (95% CI)** | **P-value*** | **Estimated ratio (95% CI)** | **P-value*** |
| Month 1 vs Baseline | 0.01 (0.00, 0.03) | <.0001 | 0.01 (0.00, 0.25) | 0.0066 |
| Month 6 vs Baseline | 0.00 (0.00, 0.00) | <.0001 | 0.02 (0.00, 0.26) | 0.0029 |
| Month 12 vs Baseline | 0.00 (0.00, 0.00) | <.0001 | 0.00 (0.00, 0.02) | <.0001 |
| Month 6 vs Month 1 | 0.02 (0.00, 0.16) | 0.0003 | 2.69 (0.05, 138.67) | 0.6201 |
| Month 12 vs Month 1 | 0.02 (0.00, 0.17) | 0.0004 | 0.15 (0.00, 8.00) | 0.3458 |
| Month 12 vs Month 6 | 1.06 (0.19, 5.85) | 0.9480 | 0.06 (0.00, 1.51) | 0.0855 |
| *: P-values were from Type III analysis based on a linear mixed-effect model. | | | | |
